# An initial experience of using dual energy contrast enhanced mammography at a tertiary care hospital in Pakistan

**DOI:** 10.12669/pjms.39.6.7607

**Published:** 2023

**Authors:** Sana Zeeshan, Gulnaz Shafqat, Danish Ali, Lubna Vohra

**Affiliations:** 1 Sana Zeeshan, MBBS, FCPS, FACS, MRBS Department of Surgery Aga Khan University Hospital (AKUH), Karachi, Pakistan; 2 Gulnaz Shafqat, MBBS, MCPS, FCPS Department of Radiology Aga Khan University Hospital (AKUH), Karachi, Pakistan; 3 Danish Ali, MBBS Dean’s Clinical Research Fellow Aga Khan University Hospital (AKUH), Karachi, Pakistan; 4 Lubna Vohra, MBBS, FCPS, FACS Department of Surgery Aga Khan University Hospital (AKUH), Karachi, Pakistan

**Keywords:** Breast cancer, Contrast enhanced mammography, lower-middle income country, LMIC, Case Series

## Abstract

**Objective:**

Contrast enhanced mammography (CEM), a relatively new and promising modality, combines mammography (MMG) with an iodinated contrast material to illuminate neovascularity within the breast; analogous to magnetic resonance imaging (MRI). CEM improves the overall sensitivity of MMG; reduces the need for unnecessary biopsies and follow-up imaging and can be considered a reasonable substitute for MRI. In Pakistan, CEM was recently introduced and to assess its usability a study was conducted on five patients before making it available as a regular investigation.

**Case presentations:**

Four out of the five patients had a clinical suspicion of malignancy with two patients having heterogeneously dense breasts and two with dense breasts. All enhancing lesions were concordant on biopsy and had similar corresponding findings on additional imaging such as ultrasound (US) and/or MRI. CEM in all four cases of biopsy proven malignancy facilitated surgical planning. The fifth patient underwent CEM for screening and was found to have no enhancing lesion.

**Conclusion:**

In low-middle-income countries (LMICs) where breast MRI is not readily available and expensive for the populace, CEM can be a reliable alternative. The initial experience with CEM at our hospital shows better visualization of malignant lesions in dense and heterogeneously dense breasts with an easy-to-perform technique and a shorter imaging time while facilitating surgical decision-making in terms of breast conservation.

## INTRODUCTION

 Breast cancer (BC) is the most commonly diagnosed cancer in Pakistan and is the leading cause of cancer-related mortality among women.^1^ Hailing a high accuracy (sensitivity of 81% and a specificity of 96%), mammography (MMG) is the most used population-based screening modality for BC; this is also the modality that is known to reliably reduce mortality amongst females aged 39 to 69 years.^2^,^3^ However, its sensitivity falls in patients with dense breasts.^3^ The other modalities commonly used for breast imaging include ultrasound (US), magnetic resonance imaging (MRI), and digital breast tomosynthesis (DBT).

 Contrast enhanced mammography (CEM), a relatively new and promising modality that combines MMG with an iodinated contrast material to illuminate neovascularity within the breast analogous to MRI,^4^ was commercially introduced in 2011.^5^ The technique is comparable to MRI and US and is superior to MMG in imaging breast diseases in different clinical vignettes.^6^ Owing to the leaky nature of neovessels, the contrast-medium diffuses in the tumor tissue and allows better visualization of the malignant tumor even in patients with dense breasts.^5^ The United States Food and Drug Administration (FDA) has officially permitted the use of CEM by allowing CEM “and/or ultrasound exams to localize a known or suspected lesion”.^7^

 CEM improves the overall sensitivity of MMG to 97% (93-100%) and the specificity to 70% (63-88%).^5^,^8^ It also improves screening amongst patients with an intermediate risk (15-20% lifetime risk) of developing BC.^5^ With superior sensitivity and negative predictive values (NPV) in assessing architectural distortion in breast parenchyma, CEM imaging can help prevent unnecessary biopsies and follow-up imaging.^5^ It can be considered a reasonable substitute for MRI, however, it does not allow adequate assessment of the axilla or other regional nodes.^5^ Nevertheless, once the primary tumor is evaluated, CEM can help in assessing whether the disease is limited to one breast only or is bilateral, unifocal, multifocal, or even multicentric.^5^

 In Pakistan, the available breast imaging modalities include US, MMG, DBT, and MRI. In April 2022, CEM was introduced at the Aga Khan University Hospital (AKUH), Karachi, Pakistan, and was initially performed on five patients to evaluate its usability in our setting before making it available as a regular investigation. This is the first time CEM has been introduced in Pakistan and this case series details our initial experience with CEM for the evaluation of different breast pathologies.

## METHODS

 We intended to recruit female patients presenting to AKUH breast clinics with a breast lump, no history of allergy to contrast agents, and a normal serum creatinine level. Approximately 20 patients were informed about CEM after they were seen and their further workup was finalized by the primary breast surgeons at AKUH, a 560-bed tertiary care hospital. All these patients were already advised for a bilateral MMG and breast US for various indications. Five patients consented to CEM after being counseled about its purpose, benefits, and adverse reactions to contrast agents such as hypotension or hypertension, tachycardia or bradycardia, bronchospasm, facial edema, laryngeal edema, pulmonary edema, or seizures. No monetary benefits were given to any patient.

 As this imaging was to establish the usability of the tool, the institution covered all monetary costs incurred and agreed to provide coverage in case of any possible adverse events due to the intravenous contrast. Hence, the examination was free of cost for all five patients. A written informed consent was taken after the participants understood the procedure, procedural benefits, and risks (Annexure-1 and Annexure-2). The participants were assured that all patient data will be de-identified before publishing and that their identity will not be disclosed to any person apart from the research team. Following the AKUH data retention policy, all the data obtained for the study will be retained for seven years on a password-protected hard disk.

### Ethical Approval:

The study has been approved by AKUH ethical review committee registered with their number 2022-7980-22747 and the research was carried out per the Declaration of Helsinki (1964).

### CEM Procedure:

Before the CEM examination, all participants were inquired about allergies and allergic reactions to the iodinated contrast agent in conformation with the AKUH guidelines.^9^ A serum creatine level was obtained in patients above the age of 50, those with a history of a renal disorder, diabetes, and/or multiple myeloma^9^ (Annexure-2). Patients with no previous history of allergies and allergic reactions to iodinated contrast and normal serum creatinine level were briefed on the risks and benefits of the examination and a written informed consent was obtained. An intravenous line was then maintained, and a low osmolar non-ionic iodinated contrast material was injected at a dose of 1-2mL/kg of body weight (maximum of 150 ml) using a power injector, for a rate of 2-3 mL/s trailed by a saline flush. Patients using metformin were asked to withhold the medication up to 48 hours post-procedure.

 CEM was performed using the standard mammographic equipment at AKUH: the GE senographe Pristina™ system. This machine has a dual-energy system that permits dual-energy subtractions to obtain a CEM image. The breast was compressed in a usual fashion akin to routine mammography and the dual-energy images were acquired in the standard views: mediolateral oblique (MLO) and craniocaudal (CC) projection of either side. Each view was comprised of two exposures. The low-energy image (25-34kv akin to full-field digital mammography) was followed rapidly by high-energy images (45-49kv). High-energy images were used to produce an iodine image (or a recombined image) that delineated the area/s of enhancement. The total force applied for breast compression was 5-8 daN (decanewton). A complete examination took 5-6 minutes to perform.

## CASE PRESENTATIONS

### Case-1:

**Fig.1:**
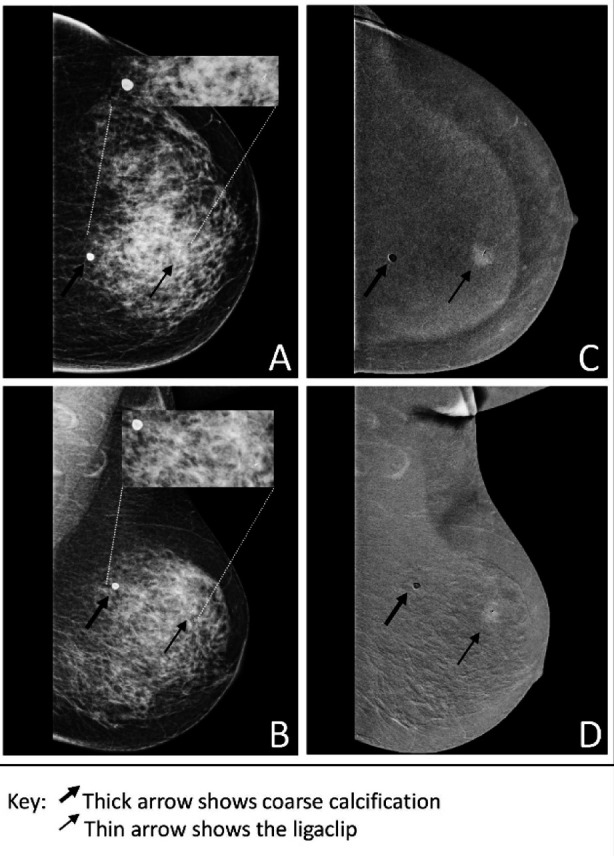
(A & B): Pre-contrast craniocaudal (CC) and mediolateral oblique (MLO) views of the left breast showing a speck of coarse calcification (thick arrow) and post-biopsy ligaclip in the upper inner quadrant (thin arrow) with no visible lesion. Inset: magnified views of breast area showing a ligaclip with coarse calcification. (C & D): Post-contrast recombined CC and MLO views show a single, well-enhancing spiculated lesion with ligaclip within it.

A 60 year old female, nulliparous, hypertensive, and diabetic, with a positive family history of BC, presented with a 2 x 2 cm ill-defined mass at the 11 o’clock position of the left breast. MMG revealed heterogeneously dense breast parenchyma without any obvious lesion. US showed a solid and lobulated suspicious mass with minimal vascularity measuring 23 x 13 x 19 mm at the 10 o’clock position, labeled as BIRADS-V, with benign-appearing axillary lymph nodes. Trucut biopsy with clip placement showed an invasive carcinoma with no special type (NST) grade-II, strong ER and PR positivity, Her-2 neu negativity, and a Ki-67 of 40%. She was initially started on hormonal treatment (letrozole). Since no obvious lesion was visualized on the MMG, an MRI breast was ordered but couldn’t be performed due to the unavailability of service. Hence, CEM was used to visualize the single lesion. Fig.1

 Pre-contrast images exhibited a post-biopsy ligaclip in the upper inner quadrant with no definite lesion. Fig.1 A, B. Post-contrast recombined images revealed a single soft tissue density measuring 14 x 12 mm in the upper inner quadrant with a biopsy clip in place showing post-contrast enhancement (Fig.1 C,D), along with a speck of benign-appearing coarse calcification. The patient underwent a left breast oncoplastic wide local excision with sentinel lymph node biopsy. The histopathology report showed a tumor bed of 1.6 x 1.5 x 1 cm with a single focus of invasive carcinoma measuring 0.9 x 0.4 cm and 5% ductal carcinoma in situ (DCIS) with up to 60% reduction in tumor cells owing to the neoadjuvant hormonal treatment administered. All margins and sentinel lymph nodes were tumor-free.

### Case-2:

**Fig.2:**
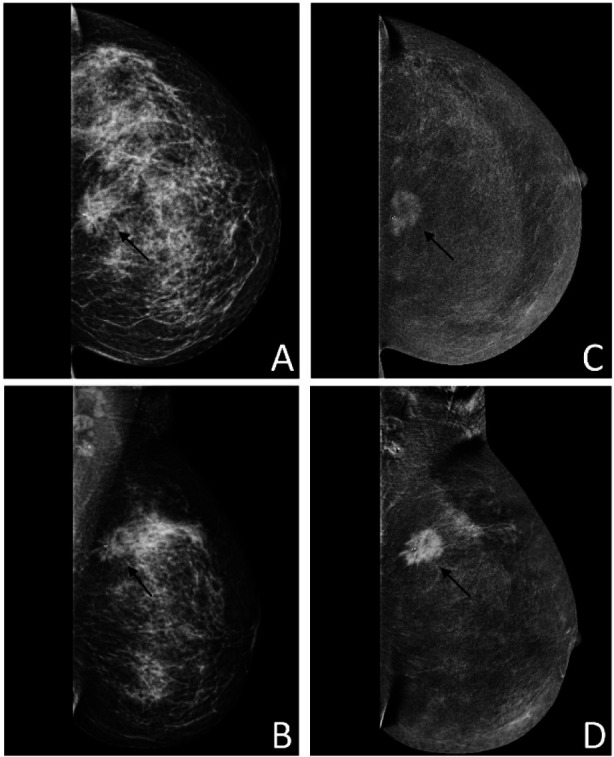
Pre-contrast (A&B) and post-contrast recombined images (C&D) of left breast demonstrating the spiculated lesion containing post-biopsy ligaclip (thin arrow). Note the clearly demarcated enhancing lesion in post-contrast images.

A 48 year old hypertensive premenopausal lady with a strong family history of BC, presented with a year old left sided breast lump. Clinically, there was a 4.5 x 5 cm palpable lump in the left breast at the 12 o’clock position. MMG showed heterogeneously dense breast parenchyma with a spiculated lesion in the upper inner quadrant of the left. The lesion had an indistinct anterior margin merging into breast parenchyma. In US, a suspicious BIRADS IV solid mass was seen at 11 o’clock measuring 24 x 19 x 16 mm. Trucut biopsy revealed invasive breast carcinoma NST grade-II, strongly positive ER, and PR, and a 3+ Her2-neu. Her CEM showed a single ill-defined density with infiltration into the surrounding breast parenchyma measuring 29 x 26 mm. Fig.2 She is currently undergoing neoadjuvant chemotherapy and targeted therapy intending to conserve the breast.

### Case-3:

**Fig.3:**
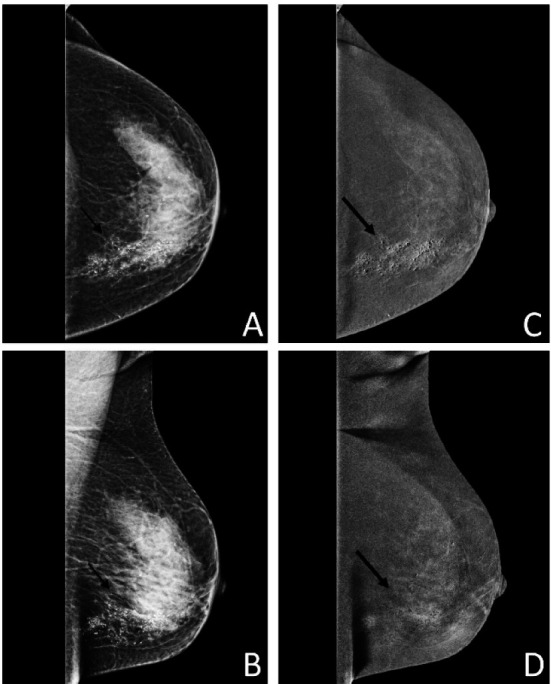
(A & B) Pre-contrast CC and MLO views of left breast showing extensive areas of microcalcifications (thin arrows). (C & D) Post-contrast recombined images of the left breast demonstrate segmental enhancement around the calcifications in the lower inner quadrant (thick arrows).

A 63 year old postmenopausal, hypertensive, diabetic lady, presented with suspicion of left breast Paget’s disease. Her breast parenchyma was dense on MMG with extensive pleomorphic microcalcifications in the left lower inner and upper outer quadrants. Segmental enhancement was seen in the lower inner quadrant in post-contrast images at the site of pleomorphic microcalcifications (Fig.3). An area of multiple echogenic foci and posterior shadowing was seen at the 7 o’clock position of the left breast on US corresponding with the area of pleomorphic calcifications seen on the MMG. Biopsy of microcalcifications revealed DCIS and the nipple wedge biopsy confirmed Paget’s disease. Final histopathology after mastectomy confirmed extensive DCIS.

### Case-4:

**Fig.4:**
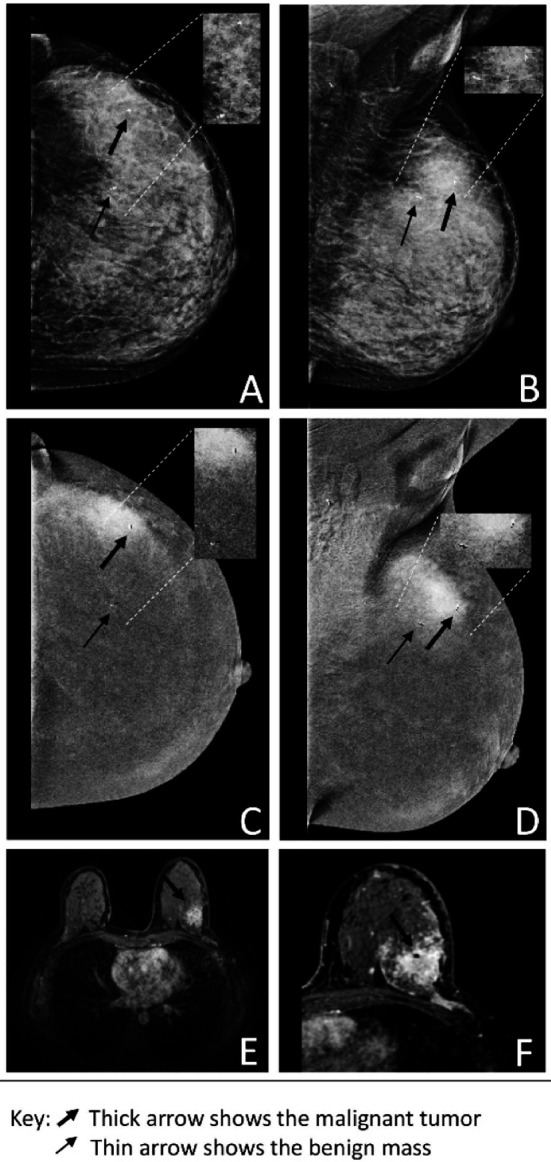
Low energy imaging CC and MLO view of the left breast (A & B). Recombined image CC and MLO view of the left breast (C & D) demonstrating biopsy-proven malignant lesion with a clip at 2 o’clock (upper outer quadrant) showing enhancement and a biopsy-proven benign lesion at 12 o’clock with the clip showing no enhancement. MRI of the left breast (E & F) shows an area of enhancement in the upper outer quadrant with a signal void area along with a post-biopsy clip.

A 36 year old lady with a strong family history of breast carcinoma, presented with a left breast lump in the upper outer quadrant measuring 3.5 x 3cm. Her breasts were dense as seen on the MMG. US identified a 33 x 28 x 17mm lesion at 2 o’clock with another lesion at 12 o’clock measuring 8 x 7mm. A biopsy of the 2 o’clock lesion showed triple negative invasive ductal carcinoma and the lesion at 12 o’clock lesion turned out to be a fibroadenoma. CEM revealed similar findings with post-contrast enhancement at the 2 o’clock lesion and no enhancement in the 12 o’clock lesion (Fig.4 A-D). Her complimentary breast MRI corresponded to CEM findings and biopsies (Fig.4 E,F).

### Case-5:

**Fig.5:**
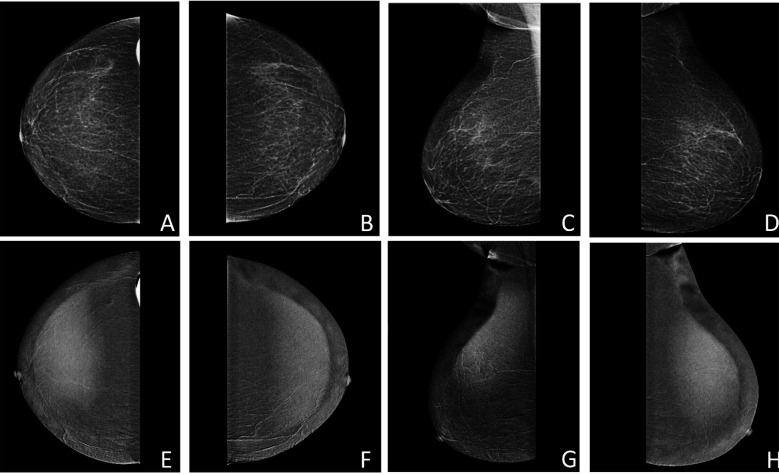
Pre-contrast (A, B, C, D) and post-contrast recombined images (E, F, G, H) of both breasts demonstrating normal breast parenchyma with no suspicious findings.

A 68 year old nulliparous, hypertensive, postmenopausal lady, presented with left mastalgia. There was a strong family history of BC. Her breast examination was unremarkable, and she was referred for BC screening. MMG showed a fibroglandular breast parenchyma with scattered specks of benign-appearing calcifications bilaterally. Her CEM did not identify any enhancing lesion which was further confirmed by US, however, some background parenchymal enhancement was seen bilaterally (Fig.5).

## DISCUSSION

 Currently, contrast enhanced breast MRI is considered the most sensitive modality for BC detection and local staging.^10^ The high sensitivity of MRI is because of the extravasation of contrast from the leaky vessels of tumoral neoangiogenesis, hence, resulting in enhancement.^5^ Using the same pathophysiology, CEM has emerged as a new technology in the field of breast imaging since the year 2000. The results were promising despite increased radiation dose and misregistration artifacts.^11^ In 2011, a dual-energy system came into existence with radiation dose within the limits, as recommended by Mammography Quality Standards Act (MQSA) and approved FDA as a diagnostic exam.^7^ This has helped in eliminating misregistration artifacts while performing whole scan of each breast in a single setting.^12^ Since then, multiple studies have been conducted to see the utility and cost-effectiveness of this new technique.

 CEM is particularly beneficial in conditions where a breast MRI would be ideally advised, such as evaluation in dense breasts for identification of lesions and multicentricity, response assessment after neoadjuvant chemotherapy, to look for residual or recurrent disease, and screening for BC in intermediate and high-risk women.^13^ A study conducted by Tennant et al.^14^ showed that sensitivity dramatically increased to 94.5% from 84.4% when CEM was added with full film digital mammography (FFDM) in symptomatic breasts. Patel et al^15^ conducted a study on 65 patients with invasive BC comparing CEM and MRI after neoadjuvant systemic therapy.

 They found similar levels of sensitivity and positive predictive values (PPVs) for showing residual disease. The study outcome suggested that CEM may be used to describe disease extent and determine treatment response. A study conducted by Iotti V et al. proved that CEM was better than MRI for predicting pathologic complete response (Lin coefficient 0.81 vs 0.59).^16^ The amount of residual disease was slightly undervalued with both radiological investigations, with CEM assessing more accurately than MRI (mean underestimation, 4.1 mm with CEM vs 7.5 mm with breast MRI).^16^

 In our study, 4 out of 5 cases of CEM had clinical suspicion of malignancy. Two patients had heterogeneously dense breasts and two had dense breasts. All enhancing malignant lesions were concordant on biopsy and had similar corresponding findings on additional imaging such as US and MRI. CEM in all four cases facilitated the surgeons in terms of surgical planning, one patient underwent mastectomy for extensive DCIS while the remaining three are for breast conservation. The fifth case was performed for screening in which no enhancing or suspicious lesion was seen in breasts with fibroglandular parenchyma. However, some background parenchymal enhancement was noted on CEM bilaterally which we attribute to technical errors since it was one of the first few cases performed when there was limited technical expertise. None of our patients had any adverse events.

 In low-middle-income countries (LMICs) such as Pakistan, where breast MRI is not readily available and expensive for the general population, CEM can be a reliable alternative. It is quick to perform, cost-effective as compared to MRI, improved sensitivity than conventional FFDM, short learning curve for radiologists and technicians, and lacks the use of a potentially claustrophobic machine.^17^ In addition, it is useful in situations when an MRI cannot be performed such as the presence of metallic foreign bodies or devices, obese patients exceeding table weight limits, and specific body habitus.^17^ It can easily be adapted and established in centers where mammographic facilities are available. In 2017, Mayo Clinic in Arizona calculated that the CEM costs a quarter of getting an MRI.^18^ In our institution, CEM costs less than a fifth of the cost of an MRI.

 Drawbacks of CEM include hypersensitivity reactions to low osmolality iodinated contrast media (approximately 4 in 10,000 administrations, with the mortality reported as 1 in 100,000-170,000 administrations),^19^ rarely reported contrast-induced nephropathy, an increased dose of radiation between 1.2 to 1.8 times of a standard FFDM (though it is still less than the recommended guidelines of 3mGy average glandular dose exposure for breast imaging)^20^, and unavailability of CEM guided system for biopsy of suspicious enhancement.^17^ Another limitation is the evaluation of axilla like standard MMG requiring a US.^5^

 Since CEM is not available in any other region of Pakistan and our hospital is the first to initiate this service, this case series is the first study on CEM being reported from our part of the world. This will help us in refining our practices for better patient care and plan future research for better utilization of this modality.

## CONCLUSION

 The initial experience with CEM at our hospital shows better visualization of malignant lesions in dense and heterogeneously dense breasts with an easy-to-perform technique and a shorter imaging time. With limited MRI facilities, it is safe to accept CEM as a viable alternative to MRI breast in LMICs like Pakistan. Our initial experience will not just help in improving our performance but will also provide a direction for other institutions in adapting and establishing this modality. Auditing and further research are required in setting up our institutional guidelines for improved quality and utility of CEM.

### Authors Contribution:

**SZ** and **GS:** Had substantial contributions to the conception and design of the work. They also played a role in drafting the work.

**DA:** Played a significant role in drafting the manuscript.

**LV**, **SZ** and **GS:** Also helped to acquire data, revise the work critically and gave the final approval of this version to be published.
